# Accounting for the temperature dependence of ^13^C spin–lattice relaxation of methyl groups in the glycyl–alanyl-leucine model system under MAS with spin diffusion

**DOI:** 10.1007/s10858-019-00261-5

**Published:** 2019-08-12

**Authors:** Van C. Phan, Elizabeth A. Fry, Kurt W. Zilm

**Affiliations:** 1 Natural Science Department, Hostos Community College, 500 Grand Concourse, The Bronx, NY 10451 USA; 2 Department of Chemistry, Yale University, P.O. Box 208107, New Haven, CT 06520-8107 USA

**Keywords:** Methyl rotation barrier, NMR relaxation, ^13^C–^13^C spin diffusion, MAS, Solid state NMR

## Abstract

The difficulties in quantitatively modeling the temperature dependence of spin–lattice relaxation in a model isotope-enriched peptide are explored as a prelude to obtaining dynamics parameters for motions in proteins from such measurements. The degree to which this can be handled by adding spin diffusion to a bath in standard rate matrix relaxation theory is studied using a small tri-peptide model system, glycyl–alanyl-leucine (GAL). We observe in this molecule that the relaxation of backbone carbons CO and Cα is not dominated by local fluctuations of the ^13^C–^1^H dipolar couplings, but rather by ^13^C–^13^C spin diffusion to nearby methyl relaxation sinks. A treatment of the methyl relaxation itself, which ignores ^13^C–^13^C spin diffusion effects back to the otherwise slowly relaxing bath, provides poor agreement between theory and experimental data obtained for the temperature dependence of the methyl relaxation rates. Closed form approximate spectral densities and relaxation rates for a methyl group during magic angle spinning are obtained to compute the needed transition rates. These average computed rates, in conjunction with an extended form of the Solomon equations, are found to adequately model the temperature dependence of the methyl relaxation rates when spin diffusion is included. The barrier to rotation for the alanine methyl in GAL is determined to be 3.5 kcal mol^−1^.

**Electronic supplementary material:**

The online version of this article (10.1007/s10858-019-00261-5) contains supplementary material, which is available to authorized users.

## Introduction

It is increasingly recognized that molecular dynamics are as relevant as molecular structure in determining the function of biologically important macromolecules (Kempf and Loria 2003). Solid state NMR (ssNMR) has several unique attributes which suit it to the study of internal dynamics in such biological systems via NMR relaxation measurements. In crystalline samples the overall rigid body tumbling which dominates solution NMR relaxation phenomena is quenched, and therefore internal and overall degrees of freedom do not have to be experimentally distinguished. The use of solid samples also makes a much wider range of temperatures feasible, potentially providing greater accuracy in determining the activation parameters associated with internal motions.

While ssNMR has become increasingly common in structural biology (Castellani et al. 2002; Zech et al. 2005), dynamical studies of proteins and other macromolecules are less prevalent. Before the advent of high resolution 2D and 3D magic angle spinning (MAS) NMR methods for solid proteins, ssNMR studies of relaxation in biochemically relevant systems required the use of site specific labels, for example the use of deuterium relaxation experiments to determine the barrier to rotation in methyl groups (Batchelder et al. 1983). More recently, site specific measurements have become possible without the need for site specific labeling. Progress has been made in using amide ^15^N relaxation to probe backbone dynamics (Chevelkov et al. 2007, 2008; Giraud et al. 2005, 2006, 2007), and in using ^2^H relaxation studies of methyl groups to study side chain dynamics (Reif et al. 2006; Tugarinov and Kay 2006). Others have used cross polarization dynamics to obtain side chain order parameters (Lorieau and McDermott 2006a, b).

Although MAS ssNMR methods have many attractive attributes, the solid state also brings with it additional complications. As noted in early work by Torchia and Szabo, T&S (1982), later addressed by Akasaka et al. (1983) and Naito et al. (1983) and more recently by Giraud et al. (2005), relaxation rates are anisotropic in solid samples, potentially complicating the analysis of the experimental data. In addition, typical samples have extensive isotopic enrichment to facilitate the use of ^1^H–^15^N, ^13^C–^15^N or ^13^C–^13^C 2D spectra to provide site specific resolution. In such samples, homonuclear spin diffusion is expected to play an important role in determining relaxation dynamics.

In this paper we examine how well we can quantitatively account for the relaxation of a specific ^13^C site while dealing with the complications of having a powder sample, MAS and extensive ^13^C enrichment. A small peptide was chosen for study as a model of expected behavior in proteins. We specifically investigate what is needed to extract dynamical parameters from ^13^C relaxation measurements of methyl groups under MAS made over a wide range of temperatures. Since it is expected that the methyl group rotation will be well described as a thermally activated process, systematic variation of the rotational correlation time, $\tau_{R}$, provides a good test of any quantitative model of the relaxation dynamics under MAS. Methyl group rotation is expected to provide a favorable case to study in that the internal dynamics are simple, well understood, and should dominate the observed relaxation rates. We demonstrate here that with the appropriate average transition rates for MAS it is possible to quantitatively reproduce the methyl group ^13^C relaxation behavior over a 140 °C temperature range with only two adjustable parameters as long as the methyl group geometry is assumed to be the same as observed by solution NMR. With the inclusion of substantial cross relaxation by spin diffusion to other ^13^C nuclei, relaxation is also modeled well for extensively ^13^C enriched samples.

## Theoretical model

Our starting point is to assume the spin–lattice relaxation for a methyl group will be described by the Solomon equations as formulated by Batchelder et al. (1983) and Macura and Ernst (2002). Defining the departures of the z-components of the magnetization from equilibrium by $m_{k} = M_{Z}^{k} (t) - M_{Z}^{k} (\infty )$:

1 $$ \frac{d}{dt}\left( {\begin{array}{c} {m_{I} } \\ {m_{S} } \\ \end{array} } \right) = - \left( {\begin{array}{cc} {R_{II} } & {R_{IS} } \\ {R_{SI} } & {R_{SS} } \\ \end{array} } \right) \cdot \left( {\begin{array}{c} {m_{I} } \\ {m_{S} } \\ \end{array} } \right). $$

Following detailed balance, the required rates can be expressed as functions of the relevant transition probabilities (Macura and Ernst 2002):

2 $$ \begin{aligned} R_{II} & = 2(n_{I} - 1)\left( {W_{1}^{II} + W_{2}^{II} } \right) + n_{S} \left( {W_{0}^{IS} + 2W_{1I}^{IS} + W_{2}^{IS} } \right), \\ R_{IS} & = n_{I} \left( {W_{2}^{IS} - W_{0}^{IS} } \right), \\ R_{SS} & = 2(n_{S} - 1)\left( {W_{1}^{SS} + W_{2}^{SS} } \right) + n_{I} \left( {W_{0}^{IS} + 2W_{1S}^{IS} + W_{2}^{IS} } \right), \\ R_{SI} & = n_{S} \left( {W_{2}^{IS} - W_{0}^{IS} } \right), \\ \end{aligned} $$

where $n_{I}$ and $n_{S}$ are the number of I (^1^H) and S (^13^C) spins in the system, respectively. Although this treatment of the transition rates incorrectly deals with potential cross-correlation effects, this can be reasoned to be minimal in the particular case studied here, a point we will return to later. The general solution to the coupled differential equations (1) is:

3 $$ \begin{aligned} m_{I} & = C_{1} Ae^{{\lambda_{1} t}} + C_{2} Be^{{\lambda_{2} t}} , \\ m_{S} & = C_{1} e^{{\lambda_{1} t}} + C_{2} e^{{\lambda_{2} t}} , \\ \end{aligned} $$

where the amplitudes $A$ and $B$ and the eigenvalues $\lambda_{1}$ and $\lambda_{2}$ are given by

4 $$ \begin{aligned} \lambda_{1} & = \frac{1}{2}\left( { - R_{II} - R_{SS} - \sqrt {R_{II}^{2} + 4R_{IS} R_{SI} - 2R_{II} R_{SS} + R_{SS}^{2} } } \right), \\ \lambda_{2} & = \frac{1}{2}\left( { - R_{II} - R_{SS} + \sqrt {R_{II}^{2} + 4R_{IS} R_{SI} - 2R_{II} R_{SS} + R_{SS}^{2} } } \right), \\ A & = - \frac{{ - R_{II} + R_{SS} - \sqrt {R_{II}^{2} + 4R_{IS} R_{SI} - 2R_{II} R_{SS} + R_{SS}^{2} } }}{{2R_{SI} }}, \\ B & = - \frac{{ - R_{II} + R_{SS} + \sqrt {R_{II}^{2} + 4R_{IS} R_{SI} - 2R_{II} R_{SS} + R_{SS}^{2} } }}{{2R_{SI} }}. \\ \end{aligned} $$

The constants $C_{1}$ and $C_{2}$ are determined by the initial conditions $m_{I} (0)$ and $m_{S} (0)$:

5 $$ \begin{aligned} C_{1} & = m_{S}(0) - \frac{{m_{I} (0) - Am_{S} (0)}}{B - A}, \\ C_{2} & = \frac{{m_{I} (0) - Am_{S} (0)}}{B - A}. \\ \end{aligned} $$

For a ^13^C saturation recovery experiment, $m_{I} (0) = 0$ and $m_{S} (0) = - M_{Z}^{S} (\infty )$ giving

6 $$ C_{1} = - M_{Z}^{S} (\infty )\left( {1 - \frac{A}{B - A}} \right)\quad C_{2} = M_{Z}^{S} (\infty )\left( {\frac{A}{B - A}} \right). $$

It is clear that the relaxation of each species of this spin system is non-exponential unless the spins are completely decoupled from one another. Furthermore, the preparation of the I spin can significantly affect the relaxation behavior of the S spin.

However, this simple model is not able to quantitatively reproduce the experimental data for ^13^C methyl group relaxation with any physically reasonable parameters in solid samples having other ^13^C enriched sites (vide infra). As a minimal treatment, these other additional ^13^C will be treated as a bath of $n_{B}$^13^C centers which cross relax with the ^13^C methyl group of interest. The augmented system, with a third equation for the ^13^C bath collectively referred to as the B-spins, can be written in matrix form as

7 $$ \frac{d}{dt}\left( {\begin{array}{c} {m_{I} } \\ {m_{S} } \\ {m_{B} } \\ \end{array} } \right) = - \left( {\begin{array}{ccc} {R^{\prime}_{II} } & {R^{\prime}_{IS} } & {R^{\prime}_{IB} } \\ {R^{\prime}_{SI} } & {R^{\prime}_{SS} } & {R^{\prime}_{SB} } \\ {R^{\prime}_{BI} } & {R^{\prime}_{BS} } & {R^{\prime}_{BB} } \\ \end{array} } \right) \cdot \left( {\begin{array}{c} {m_{I} } \\ {m_{S} } \\ {m_{B} } \\ \end{array} } \right) = - {\mathop R\limits_{ \approx }}^{\prime} \cdot \overset{\lower0.5em\hbox{$\smash{\scriptscriptstyle\rightharpoonup}$}} {m} (t). $$

In Eq. 7 the elements $R^{\prime}_{ij}$ denote that these include the effects of spin diffusion. Experimental results with a natural abundance sample finds the relaxation rate of the bath centers in the absence of ^13^C–^13^C spin-diffusion very slow, justifying setting all but the cross relaxation component of the $R^{\prime}_{BB}$ term to zero. In addition, the methyl protons have little direct effect on the relaxation of the other ^13^C sites. We can then recast the relaxation matrix in terms of the rates in the absence of spin diffusion, plus a single additional cross-relaxation rate as:

8 $$ {\mathop R\limits_{ \approx }}^{\prime}\approx \left( {\begin{array}{ccc} {R_{II} } & {R_{IS} } & 0 \\ {R_{SI} } & {R_{SS} + n_{B} \sigma } & { - \sigma } \\ 0 & { - n_{B} \sigma } & \sigma \\ \end{array} } \right). $$

In Eq. 8, $n_{B}$ is the number of isotope enriched carbons involved in the cross-relaxation and $\sigma$ is the magnitude of effective cross relaxation rate between the methyl group and the B spins. The cross relaxation rate is assumed to be negative since we expect the zero quantum rate to dominate in the slow motion limit. The solution to this system of equations will be a triple exponential of the form:

9 $$ M(t) = M_{0} \left( {1 - \alpha_{1} e^{{ - \lambda^{\prime}_{1} t}} - \alpha_{2} e^{{ - \lambda^{\prime}_{2} t}} - \alpha_{3} e^{{ - \lambda^{\prime}_{3} t}} } \right), $$

where the $\alpha_{k}$ are the amplitudes of the three relaxation rate eigenvalues $\lambda^{\prime}_{k} .$ As will be discussed later, the relative $\lambda^{\prime}_{k}$ rates encountered in practice result in the relaxation being effectively double exponential. One of the relaxation rate eigenvalues has a near zero amplitude factor and ends up not being important for analysis of the data. Of the remaining two eigenvalue*s*, one is largely determined by the geometry and motion of the methyl group, while the other reflects spin diffusion to the bath.

This framework does not include several potentially important effects. First, spin diffusion to the ^1^H bath from the methyl ^1^Hs surely occurs. However, in fitting our data, the inclusion of ^13^C spin diffusion provided a model with better agreement than inclusion of ^1^H–^1^H spin diffusion alone, and inclusion of both introduces more parameters than justified by the improvement in fit. Our calculations have also ignored the effects that ^1^H–^1^H to ^13^C–^1^H dipole–dipole cross correlation might have on the relaxation rates. It has been suggested that cross correlation can affect the measurement of correlation times by 6–15% (Cutnell and Glasel 1976). The effect of cross correlation is ordinarily to retard spin–lattice relaxation rates (Kumar et al. 2000). While these effects are expected to be more significant in the ^1^H transition rates, their presence will trickle into ^13^C T_1_ measurements through cross relaxation. For a J-resolved quartet corresponding to a methyl group, this type of cross-correlation will manifest itself as differential relaxation rates for the outer and inner peaks of the quartet. However, in the solid samples studied here, the methyl resonances do not have J-resolved multiplets, and thus only the averaged effects of the cross correlation can be measured. In such a case where net magnetization is monitored and the multiplet cannot be resolved, it has been shown that cross-correlation terms can largely be ignored as they have little effect on the accuracy of the experiment (Werbelow and Grant 1975). Thus, applying a relaxation matrix to this problem is a justifiable approximation.

To proceed further we need to compute the rates required in Eq. 8 with as few free parameters as possible. The relaxation of a methyl group is usually described by a motional model involving jumps between three symmetry related sites or involving free diffusion of the ^13^C–^1^H vector on the surface of a cone about the local C_3_ axis of the methyl group. The correlation functions for both models have been previously derived and both models yield similar results. Since the free diffusion model employs the fewest parameters, it will be adopted herein and we will closely follow the treatment by T&S (1982). Equation 48 in T&S gives the ^13^C relaxation rate for a methyl carbon with a single ^13^C–^1^H coupling and for a particular orientation of the methyl rotation axis:

10 $$ \begin{aligned} \frac{1}{{T_{1} }} & = \frac{{9\omega_{{D_{IS} }}^{2} }}{64}\{ g(\tau_{1} ,\;\omega_{I} - \omega_{s} )A_{1} B_{1} + g(\tau_{2} ,\;\omega_{I} - \omega_{s} )A_{2} B_{2} + g(\tau_{1} ,\;\omega_{S} )2A_{1} B_{4} \\ & \quad + g(\tau_{2} ,\;\omega_{S} )2A_{2} B_{5} + g(\tau_{1} ,\;\omega_{I} + \omega_{S} )4A_{1} B_{5} + g(\tau_{2} ,\;\omega_{I} + \omega_{S} )A_{2} B_{6} \} . \\ \end{aligned} $$

The rate is a function of two geometric parameters: the angle $\varTheta_{IS}$ between the methyl C_3_ axis and the ^13^C–^1^H vector, and the angle $\theta$ between the C_3_ axis and B_o_. The trigonometric functions $A_{1} = \sin^{2} 2\varTheta$ and $A_{2} = \sin^{4} \varTheta$ depend only on the local geometry, while the $B_{k}$ depend solely on ${\text{cos}}\,{\theta}$.

11 $$ \begin{aligned} & B_{1} = 4(\cos^{2} \theta - \cos^{4} \theta )\quad B_{2} = 1 - 2\cos^{2} \theta + \cos^{4} \theta \quad B_{4} = 4\cos^{4} \theta - 3\cos^{2} \theta + 1, \\ & B_{5} = 1 - \cos^{4} \theta \quad B_{6} = 1 + 6\cos^{2} \theta + \cos^{4} \theta . \\ \end{aligned} $$

The dipolar coupling is $\omega_{{D_{IS} }} = \gamma_{I} \gamma_{S} \hbar /r_{IS}^{3}$ and the spectral densities are defined as $g(\tau ,\;\omega ) = \frac{\tau }{{1 + \omega^{2} \tau^{2} }}.$ Two correlations times appear which are related by $\tau_{2} = \frac{{\tau_{1} }}{4}.$ The IS transition rates needed in Eq. 2 can be recognized in Eq. 10. Making this correspondence we have

12 $$ \begin{aligned} W_{0}^{IS} = & \frac{{9\omega_{{D_{IS} }}^{2} }}{64}\left( {g(\tau_{1} ,\;\omega_{I} - \omega_{s} )A_{1} B_{1} + g(\tau_{2} ,\;\omega_{I} - \omega_{s} )A_{2} B_{2} } \right), \\ W_{1S}^{IS} = & \frac{{9\omega_{{D_{IS} }}^{2} }}{64}\left( {g(\tau_{1} ,\;\omega_{S} )A_{1} B_{4} + g(\tau_{2} ,\;\omega_{S} )A_{2} B_{5} } \right), \\ W_{2}^{IS} = & \frac{{9\omega_{{D_{IS} }}^{2} }}{64}\left( {g(\tau_{1} ,\;\omega_{I} + \omega_{S} )4A_{1} B_{5} + g(\tau_{2} ,\;\omega_{I} + \omega_{S} )A_{2} B_{6} } \right). \\ \end{aligned} $$

These equations follow the convention adopted herein where the ^13^C is the S spin, and thus have I and S permuted in comparison to T&S. Using $\varTheta_{IS}=69.1^{\text{o}}$ and $r_{CH}=1.106\,\AA$ (Ottiger and Bax 1999) provides the relevant $W_{k}^{IS}$ as a function of B_o_ and $\tau_{1}$. Computation of the rates in Eq. 2 also requires $W_{1}^{II}$ and $W_{2}^{II} .$ For these we also use Eq. 12 with substitution of $\omega_{{D_{II} }} = \gamma_{I}^{2} \hbar /r_{II}^{3}$ and $\varTheta_{II} = 90^{o}$ for $\omega_{{D_{IS} }}$ and $\varTheta_{IS}$, respectively.

During MAS at a rate $$ \omega_{r}, {\text{cos}}\,\theta $$ becomes time dependent, and therefore so do the functions $$ B_{k} $$. It is then natural to recast the orientation of the methyl C_3_ axis in terms of its orientation in spherical polar coordinates in the MAS rotor frame. Using $$ \beta $$ as the angle between the methyl C_3_ axis and the rotor axis, and $$ \omega_{r} t + \varphi $$ as the azimuthal angle, $$ {\text{cos}}\,\theta $$ varies as a function of time as $$ \cos \theta = \sqrt {\tfrac{2}{3}} \sin \beta \cos (\omega_{r} t + \varphi ) + \sqrt {\tfrac{1}{3}} \cos \beta $$ under MAS. The rates in Eq. 7 are then time-dependent, making an exact analytic solution impractical. For a time period $$ \tau $$ short enough to consider $$ {\mathop R\limits_{ \approx }}^{\prime} $$ as constant, the longitudinal magnetization difference vector $$ \overset{\lower0.5em\hbox{$\smash{\scriptscriptstyle\rightharpoonup}$}} {m} (t) $$ formally follows $$ \overset{\lower0.5em\hbox{$\smash{\scriptscriptstyle\rightharpoonup}$}} {m} (t + \tau ) = e^{-\mathop {R^{\prime}}\limits_{ \approx }\!\tau} \cdot \overset{\lower0.5em\hbox{$\smash{\scriptscriptstyle\rightharpoonup}$}} {m} (t). $$ If $$ {\mathop R\limits_{ \approx }}^{\prime} $$ commuted with itself at all points in time during a rotor cycle, the average $$ {\mathop {\bar{R}}\limits_{ \approx }}^{\prime} = \omega_{r} \int\limits_{0}^{{1/\omega_{r} }} \mathop {R^{\prime}}\limits_{ \approx } (t)dt $$ would exactly describe the relaxation over each subsequent rotor period. Although this will not in general be true, the average $$ {\mathop {\bar{R}}\limits_{ \approx }}^{\prime} $$ should in fact be a very accurate approximation as long as the net relaxation over a rotor period is very small. In a manner similar to average Hamiltonian theory, we can then justify replacing $$ {\mathop R\limits_{ \approx }}^{\prime} $$ by its average $$ {\mathop {\bar{R}}\limits_{ \approx }}^{\prime} $$ over a rotor period since the MAS cycle and relaxation rates differ by four orders of magnitude. Related arguments were first made by T&S in anticipating the extension of their results to MAS, and have been invoked by subsequent researchers.

**Fig. 1:**
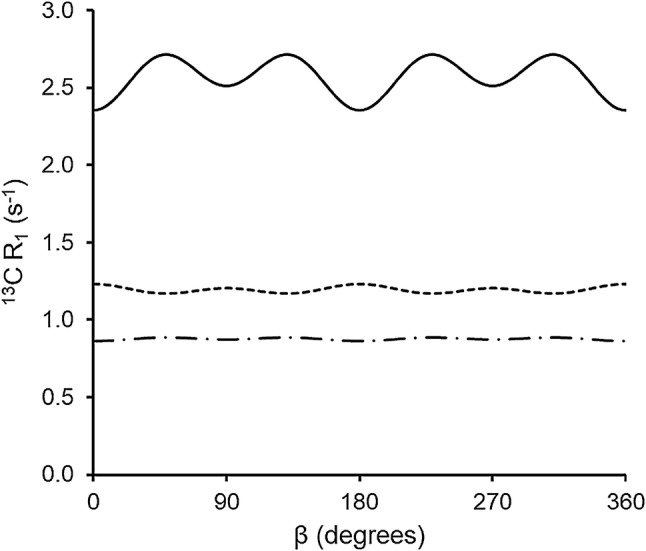
The ^13^C R_1_ calculated for a methyl group at various correlation times as a function of $\beta$. The relaxation rates were calculated using a $\varTheta$ value of 69.1° and a C–H internuclear distance of 1.106 Å. The solid curve represents the $R_{1}$ rate with a correlation time of $\tau_{1}$ = 1 × 10^−9^ s, the sort dashed curve uses $\tau_{1}$ = 1 × 10^−8^ s, and the dash dot curve has $\tau_{1}$ = 1 × 10^−10^ s

To calculate the time averaged transition rates we set $$ \cos \theta = \sqrt {\tfrac{2}{3}} \sin \beta \cos (\omega_{r} t + \varphi ) + \sqrt {\tfrac{1}{3}} \cos \beta $$ and integrate over a rotor period. The results can be obtained by substituting $$ \overline{{\cos^{2} \theta (t)}} = \frac{1}{3} $$ and $$ \overline{{\cos^{4} \theta (t)}} = \frac{1}{9}\cos^{4} \beta + \frac{2}{3}\sin^{2} \beta \cos^{2} \beta + \frac{1}{6}\sin^{4} \beta $$ into Eq. 11. The time averaged rates are then computed from Eq. 12 using the averaged $$ \bar{B}_{k} (\beta ). $$ Under MAS the relaxation will be anisotropic, with the relaxation rates dependent on the inclination $$ \beta $$ of the methyl C_3_ axis from the MAS rotor axis. On closer inspection the residual relaxation anisotropy that remains after the averaging by MAS is found to be fairly small. As an example, consider the $$ \beta $$ dependence of the ^13^C relaxation rate for a single ^13^C^1^H vector diffusing on a cone as given in Eq. 10. This rate is plotted in Fig. 1 for a ^1^H frequency of 800 MHz as a function of $$ \beta $$. The anisotropy is greatest when close to the T_1_ minimum, but even then, the variance is only ~ ±14% of the average rate. Such a small dispersion in rates would be difficult to experimentally distinguish from single exponential relaxation at the average rate. Therefore, we will also average the $$ \bar{B}_{k} (\beta ) $$ over $$ \beta $$. In computing the transition rates from Eq. 12 we then use $$ \bar{B}_{1} = \bar{B}_{2} = \frac{8}{15}, $$$$ \bar{B}_{4} = \bar{B}_{5} = \frac{4}{5}, $$ and $$ \bar{B}_{6} = \frac{16}{5}. $$ As a result, closed form expressions for the transition rates appropriate for MAS can be obtained:

13 $$ \begin{aligned} W_{0}^{IS} & = \frac{{3\omega_{{D_{IS} }}^{2} }}{40}\left( {g(\tau_{1} ,\;\omega_{I} - \omega_{s} )\sin^{2} 2\varTheta + g(\tau_{2} ,\;\omega_{I} - \omega_{s} )\sin^{4} \varTheta } \right), \\ W_{1S}^{IS} & = \frac{{9\omega_{{D_{IS} }}^{2} }}{80}\left( {g(\tau_{1} ,\;\omega_{S} )\sin^{2} 2\varTheta + g(\tau_{2} ,\;\omega_{S} )\sin^{4} \varTheta } \right), \\ W_{2}^{IS} & = \frac{{18\omega_{{D_{IS} }}^{2} }}{40}\left( {g(\tau_{1} ,\;\omega_{I} + \omega_{S} )\sin^{2} 2\varTheta + g(\tau_{2} ,\;\omega_{I} + \omega_{S} )\sin^{4} \varTheta } \right). \\ \end{aligned} $$

It should be noted that this double averaging scheme to account for the apparent lack of relaxation anisotropy under MAS was previously applied to methyl groups by Naito et al. (1983). However, in that work the relevant equations apparently contain typographical errors to the extent that we were unable to replicate their expressions for the relaxation rates. Similar averaging has been computationally studied for the relaxation of amide groups by modulation of the ^15^N–^1^H dipolar coupling by Giraud et al. (2005). While developed specifically for a freely diffusing methyl group, the expressions here can also be used to model the relaxation due to libration of an X–^1^H bond vector to the extent that diffusion on a cone at a fixed angle will replicate librational diffusion within a conical space.

## Experimental

### Sample

Three isotopomers of glycyl–alanyl-leucine (GAL) were synthesized by solid phase peptide synthesis using isotopically enriched materials purchased from Cambridge Isotope Laboratories, with methods detailed in Chen and White (2000). Samples of natural abundance GAL (naGAL), uniformly enriched in ^13^C/^15^N GAL (NCGAL) and a sample of uniformly enriched in ^2^H/^13^C/^15^N GAL (NCDGAL) were all prepared. All samples were back exchanged with water prior to crystallization; thus there are protons at all exchangeable sites.

### NMR spectroscopy

All data were acquired on a Varian Inova 800 MHz spectrometer using a home-built triple resonance (^1^H/^13^C/^15^N) CPMAS probe, employing 2.5 mm rotors with sample volumes of 6.5 μL. The MAS rate of all experiments was maintained at 19.2 kHz. For all experiments, the ^13^C power level was set to provide a radio frequency field amplitude of $\omega_{1}^{C}$/2π = 77 kHz. TPPM (Bennett et al. 1995) decoupling was implemented and a ^1^H power level $\omega_{1}^{H}$/2π = 103 kHz was employed during this period. All spectra were referenced according to previously published protocols (Morcombe and Zilm 2003).

All T_1_ relaxation experiments were performed using the saturation recovery method. Experiments were performed at 268 K, 233 K, 198 K, 178 K, 149 K and 123 K for the NCGAL sample. For the naGAL and NCDGAL sample, experiments were performed at 268 K only. The temperature was calibrated against the melting points of acetonitrile, toluene and *n*-pentane. ^13^C T_1_ relaxation curves were obtained by saturating the ^13^C spins, allowing for relaxation and applying a rotor synchronous spin echo before detection. In order to minimize ^1^H–^13^C cross relaxation during the experiment, a recycle delay of 5 × ${\text{T}}_{1}^{\text{H}}$ was implemented for all ^13^C T_1_ measurements. The methyl group data were fitted to a double exponential of the form:

14 $$ M_{z} (t) = M_{0} \left( {1 - \alpha e^{{ - \lambda_{1} t}} - (1 - \alpha )e^{{ - \lambda_{2} t}} } \right). $$

All other relaxation profiles were fit to single exponentials.

## Results and discussion

### Temperature dependence of methyl group relaxation

**Fig. 2:**
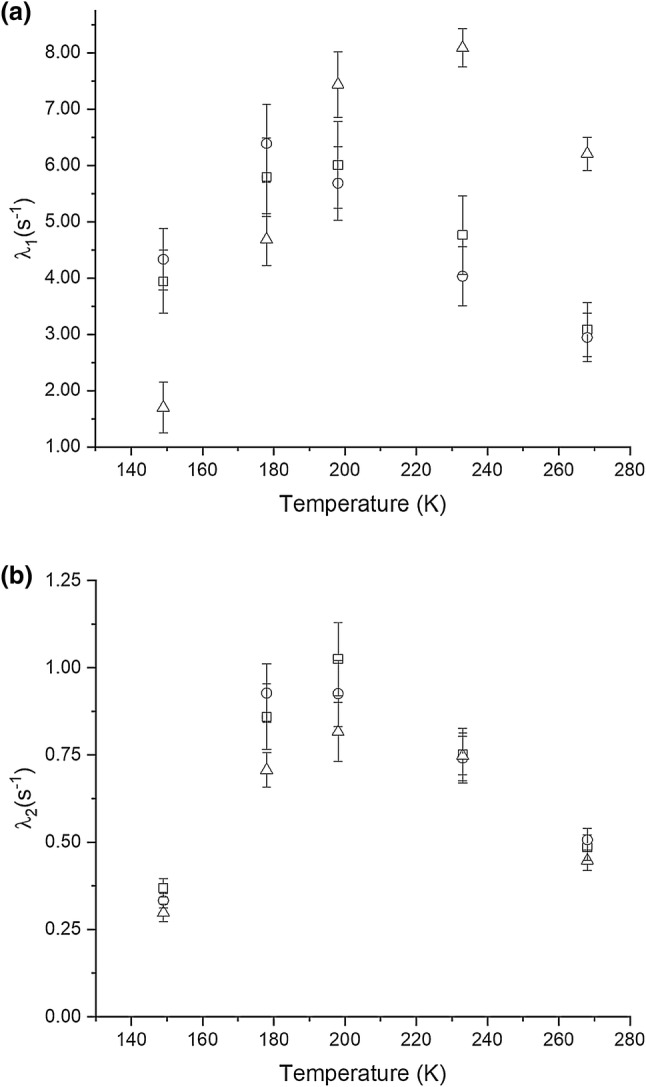
The temperature dependence of measured ^13^C relaxation rates for the methyl groups in NCGAL. The rates were fitted using the bi-exponential expression in Eq. 14, where **a** corresponds to the faster of the two measured relaxation rates and **b** corresponds to the slower of the two relaxation rates. (Open square) Represents the relaxation rates for the LCδ1 methyl group, (open circle) represents the relaxation rates for the LCδ2 methyl group, and (open triangle) represents the relaxation rates for the ACβ methyl group

The ^13^C saturation recovery data for the methyl groups in NCGAL cannot be fit by single exponentials, but are fit well by double exponential recovery curves. Figure 2 shows the temperature dependence of the fast and slow relaxation rates from such fits. The fast relaxation rate peaks at ~ 225 K for the alanine methyl and ~ 190 K for the leucine methyls. The maximum for the slow relaxation rate for all three methyl groups is at ~ 195 K. To account for the temperature dependence of the rotational correlation time, we assume this to be a temperature activated Arrhenius process. Thus we can express the correlation time $\tau_{1}$ as a function of temperature with an activation energy $E_{a}$ and a pre-exponential $\tau_{o}$:

15 $$ \tau_{1} = \tau_{o} e^{{{{E_{a} } \mathord{\left/ {\vphantom {{E_{a} } {RT}}} \right. \kern-0pt} {RT}}}} . $$

**Fig. 3:**
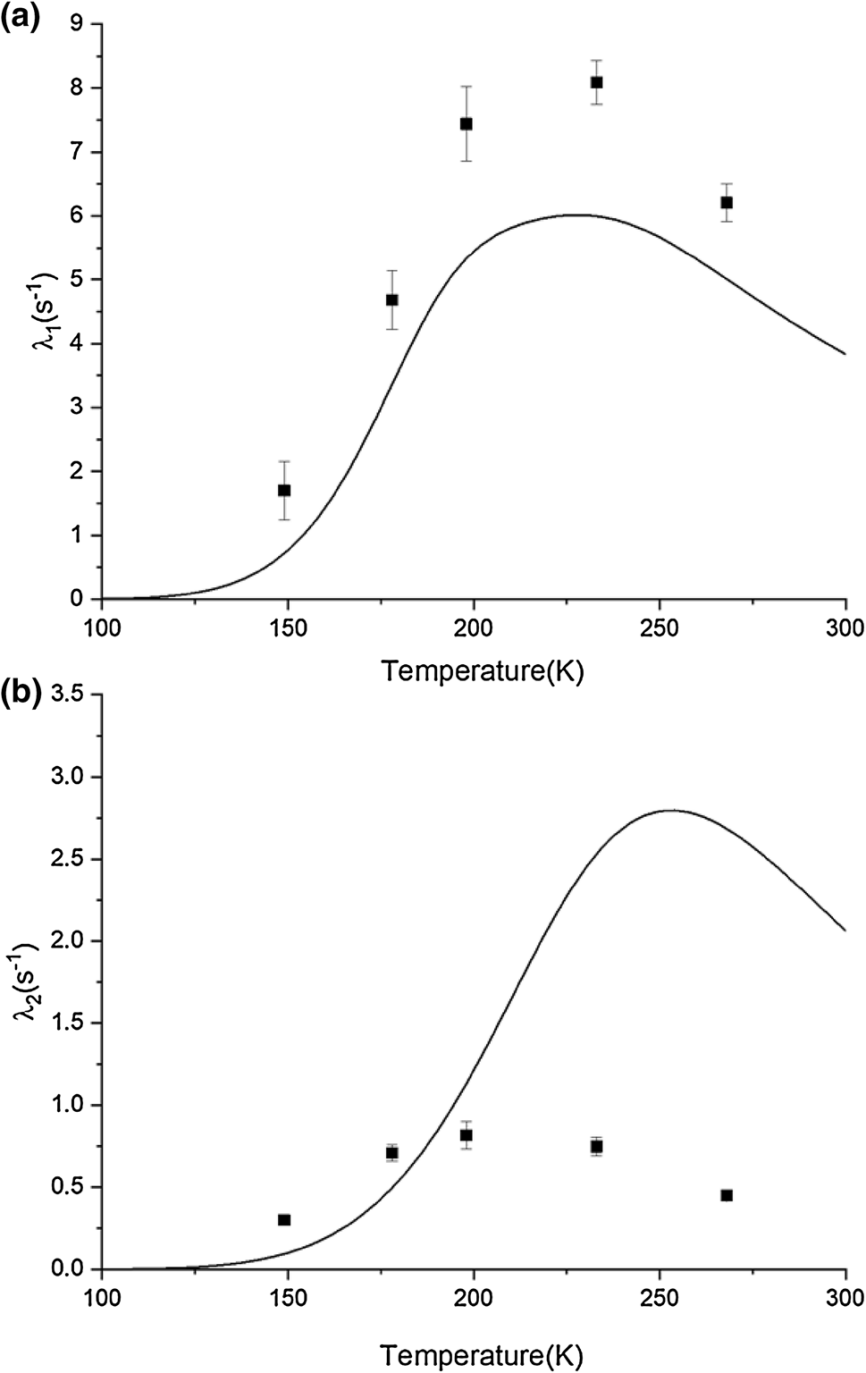
Fit of **a** the fast alanine methyl ^13^C relaxation rate and **b** the slow alanine methyl ^13^C relaxation rates to Eqs. 1–6 assuming the correlation time $\tau_{1}$ follows Arrhenius behavior as defined by Eq. 15. An activation energy of 3.05 kcal mol^−1^ and an Arrhenius prefactor $\tau_{o}$ of 1.3 × 10^−12^ s was used

If the methyl group ^13^C relaxation is sufficiently dominated by the internal ^13^C–^1^H dipolar interaction, it should be possible, using Eq. 4, to compute the two relaxation rate eigenvalues and the relative proportions of the two terms obtained from fits to Eq. 14. Figure 3 depicts the best fit that could be obtained considering only the two relaxation rates as a function of temperature. The overall temperature dependence requires an activation energy of 3.05 kcal mol^−1^ and a $\tau_{o}$ = 1.3 × 10^−12^ s. While the fast rate is somewhat accounted for, the slow rate is significantly overestimated. In addition, the relative amplitudes of the slow and fast processes are not reproduced by this simplest model. Table 1 reports the values obtained from fits of the data for the parameter $\alpha$ in Eq. 14 that gives the proportion of the fast relaxing component. Experimentally, this starts out at about 60% of the relaxation curve at the upper end of the temperature range and decreases to 22% at the low end. In contrast, the parameters that produce the best fit to the rates in the model result in a prediction that the relaxation is essentially single exponential, and nearly always dominated by the faster rate process. This is not a result of uncertainty in the fitting of the data, as all recovery curves are distinctly double exponential. The contributions of the two rates are both significant to the overall relaxation, and the rates are quite distinct. Therefore, the rates and the $\alpha$ parameter are well determined, with little interdependency encountered in the fitting. In the low temperature experiment, the slow relaxation is the dominating process, which is in clear disagreement with the predictions by the simple model. Clearly then the treatment as an isolated ^13^C^1^H_3_ spin system is inadequate.

**Table 1:** Experimental and theoretical values for the parameter α in the fitting function $M_{z} (t) = M_{0} (1 - \alpha e^{{ - \lambda_{1} t}} - (1 - \alpha )e^{{ - \lambda_{2} t}} )$

Temperature (K)	Experimental	Theoretical
268	0.58 ± 0.014	0.85
233	0.66 ± 0.014	0.96
198	0.60 ± 0.029	1
178	0.42 ± 0.034	1
149	0.22 ± 0.058	1

### Temperature dependence of backbone relaxation

**Fig. 4:**
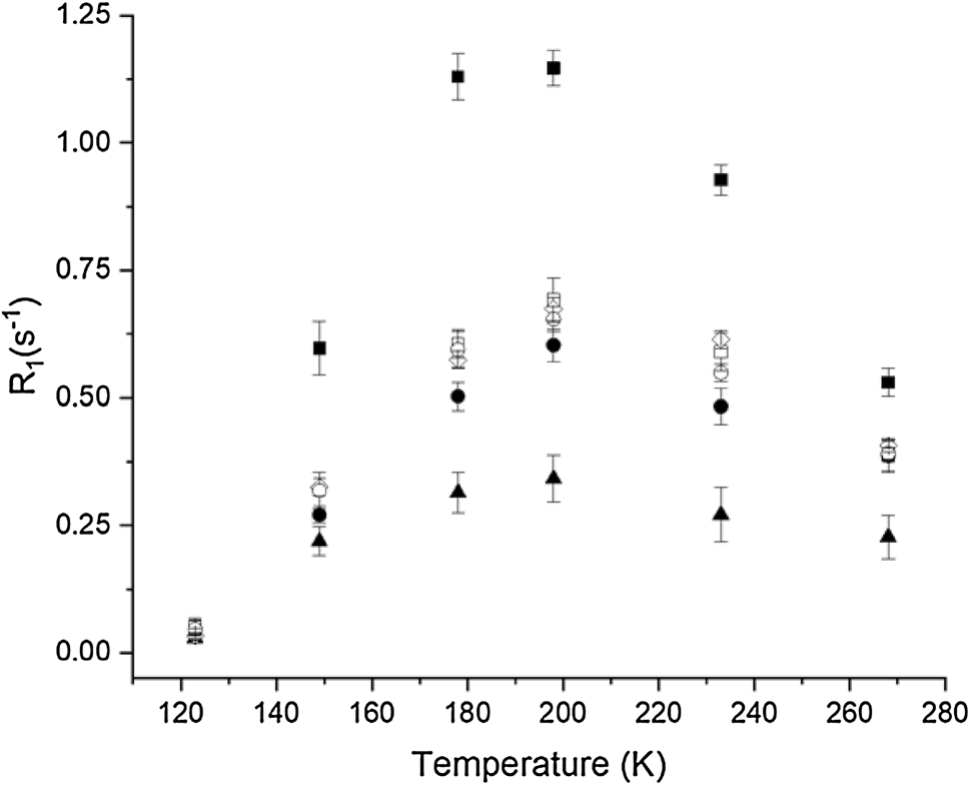
The temperature dependence of the ^13^C $R_{1}$ rates of the backbone carbon centers in NCGAL. The relaxation profiles were fit to a single exponential of the form $M(t) = M_{o} e^{{ - R_{1} t}} .$ The residues are (open square) LCO, (filled circle) ACO, (filled triangle) GCO, (filled square) LCα, (open diamond) ACα, and (open circle) GCα

Examination of the relaxation of the backbone carbon nuclei provided the first indication that inclusion of additional ^13^C spins would be required to fit the methyl group relaxation. The saturation recovery profiles for these ^13^C sites are fit very well by single exponential curves over the entire temperature range. Figure 4 depicts the temperature dependence of this single rate for the backbone CO and Cα relaxation rates. Similar to the methyl group slow relaxation rates, the backbone relaxation rates peak at ~ 195 K, indicating that the correlation times for both the backbone carbons and methyl group have slowed to the order of $1/\omega_{C}$ or the ns timescale. However, one would not expect both methyl group rotation and backbone librational motion to have the same correlation times and activation energies. Therefore, it would appear that one set of spins is serving as a relaxation sink for the other. In this case the sink will relax at a rate dominated by its internal dynamics, while the other set relaxes at essentially its cross relaxation rate to the sink, and the rates for both will appear to have the same temperature dependence.

To test this hypothesis we measured relaxation times for three different isotopomers of GAL. Table 2 compares the observed rates at 268 K for the alanine methyl site (fast relaxation rate only) and a few backbone sites. The most telling difference is between the relaxation rates for the ^13^C enriched NCGAL sample and those for a natural abundance sample (naGAL). First we notice that the fast relaxation component for the alanine methyl is very similar in both samples, being on the order of ~ 5 s^−1^. In contrast, the backbone sites could hardly be more different, with the rates in the naGAL being two orders of magnitude slower. If the relaxation for the Cα ^13^C in the NCGAL sample were due to the ^13^Cα–^1^Hα dipolar interaction, these rates would have been similar, and this is obviously not the case. The relaxation then must come from interaction with another set of spins.

**Table 2:** R_1_ rates for three isotopomers of GAL at 268 K

Sample	LCO (s^−1^)	LCα (s^−1^)	ACα (s^−1^)	GCα (s^−1^)	ACβ (s^−1^)
NCGAL	0.39	0.53	0.41	0.39	6.3
NCDGAL	0.0125	0.014	0.016	0.019	0.5
naGAL	< 0.005	< 0.005	< 0.005	< 0.005	4.5

In the NCDGAL sample we have enriched the peptide with ^13^C, and additionally replaced all the non-exchangeable protons with deuterium. Deuteration in this case reduces all the relaxation rates. If the ^13^C relaxation is by a direct ^13^C–^1^H dipolar coupling, deuteration should reduce the relaxation rate by $\frac{{\gamma_{H}^{2} I^{H} (I^{H} + 1)}}{{\gamma_{D}^{2} I^{D} (I^{D} + 1)}} \approx 16.$ In this experiment the alanine methyl relaxation is indeed reduced by a factor of 12.6, clearly indicating that the C–H dipolar interaction provides the dominant relaxation mechanism for the methyl group. While deuteration also reduces the ^13^Cα and ^13^CO relaxation rates, comparison to the naGAL sample already ruled out a C–H dipolar mediated mechanism for these sites. Both the ^13^Cα and ^13^CO rates lengthen by similar amounts, and importantly, the rates are not as slow as in the naGAL sample. This observation demonstrates the ^13^Cα and ^13^CO are both relaxed by a ^13^C–^13^C interaction with the methyl group. In the naGAL sample the ^13^C are dilute and this interaction is absent. When the sample is both ^13^C enriched and deuterated the relaxation rates slow for the backbone sites, but only because the methyl relaxation itself was reduced by the deuteration.

### Inclusion of ^13^C–^13^C spin diffusion

Since ^13^C–^13^C spin diffusion is clearly important in the relaxation of the ^13^C enriched peptide, we cannot expect to quantitatively model the relaxation of methyl groups without explicitly including this effect, and qualitatively this offers a ready explanation as to why Eqs. 3–6 do not fit the data. While we should expect the fast relaxation process to reflect the spin relaxation internal to the ^13^C^1^H_3_ group, the final recovery will be retarded by ^13^CH_3_ polarization leaking back into the slowly relaxing bath of other ^13^C nuclei. Therefore if the model is adjusted to fit the fast process, it will necessarily overestimate the slow relaxation eigenvalue if ^13^C–^13^C spin diffusion is not taken into account.

At any given temperature, the saturation recovery curves potentially will provide three rates and three amplitudes according to Eq. 9, which must be consistent with the solution of Eq. 7 and with the reduced rate matrix in Eq. 8. The elements in Eq. 8 depend upon $n_{B}, \tau$ and the rates $R_{II}, R_{IS}, R_{SI}\,{\text{and}}\,R_{SS}$, which themselves can be computed from Eqs. 2 and 13. Together the transition probabilities in Eq. 13 only depend on $\tau_{1}$ and the geometry of the methyl group. However, we expect librational motion of the methyl group CH vectors and C_3_ axis to result in reduced effective dipolar couplings, and therefore we also include an order parameter $S^{2}$ to reduce the dipolar coupling (Ottiger and Bax 1999). In principle $n_{B}, \sigma, \tau_{1}\,{\text{and}}\,S^{2}$ are then the only adjustable parameters needed to fit the relaxation curves at any particular temperature.

The best fits to the data were obtained from a binary grid search. An array of the parameters $n_{B}, \sigma, S^{2}\,{\text{and}}\,\tau_{1}$ was constructed for each temperature. These were used to compute the elements in Eq. 8, and the resulting eigenvalues and amplitudes were found by numerically solving Eq. 7. The parameters providing the best agreement at each temperature are reported in Table 3. Although the recovery curves are in principle triple exponential, the intermediate component ($\lambda_{3}$) has a very small amplitude ($\alpha_{3}$), so small in fact as to be experimentally indistinguishable from zero. This suggests that a simpler model would neglect ^13^C–^1^H cross relaxation altogether, reducing the rate matrix in Eq. 8 to the lower right 2 × 2 block. Such a model, in fact, also closely reproduces the experimental data, providing the parameters reported in Table 4. Within experimental error, the 3-spin population model relaxation rates and amplitudes $\lambda_{1}, \lambda_{2}\,{\text{and}}\,\alpha_{1}, \alpha_{2}$ are identical to those obtained from reducing the 3-spin to an effective 2-spin population model and fitting with $\lambda_{1}, \lambda_{2}\,{\text{and}}\,\alpha, 1-\alpha$.

**Table 3:** Parameters from modeling cross-relaxation among three types of spins

T (K)	$n$	$\tau$ (s^−1^)	$S^{2}$	$\lambda_{1}$ (s^−1^)	$\lambda_{2}$ (s^−1^)	$\lambda_{3}$ (s^−1^)	$\alpha_{1}$	$\alpha_{2}$	$\alpha_{3}$
268	2.7	0.70	0.93	6.3	0.45	2.9	0.60	0.39	0.0053
233	1.9	1.1	0.95	8.0	0.74	2.8	0.66	0.34	−0.0023
198	1.7	1.3	0.94	7.5	0.81	1.2	0.61	0.39	−0.0022
178	1.2	1.4	0.91	4.7	0.70	0.47	0.42	0.56	−0.0140
149	0.70	0.85	0.93	1.8	0.30	0.095	0.22	0.78	−0.0025

**Table 4:** Parameters from modeling using an effective two spin type system

T (K)	$n$	$\tau$ (s^−1^)	*S* ^2^	$\lambda_{1}$ (s^−1^)	$\lambda_{2}$ (s^−1^)	$\alpha$
268	2.9	0.73	0.92	6.0	0.44	0.58
233	2.0	1.1	0.95	8.1	0.74	0.66
198	1.7	1.3	0.94	7.4	0.82	0.60
178	1.2	1.4	0.91	4.7	0.70	0.42
149	0.70	0.83	0.93	1.7	0.30	0.23

The best fits require a physically reasonable methyl libration order parameter in the range of 0.93 ± 0.02. This value agrees very well with the results from Ottiger and Bax (1999) obtained in liquid crystal NMR studies, and is independent of the other fitting parameters. The values of $n_{B}$ and $\sigma$ vary more systematically with temperature, and are somewhat interdependent. Except at the lowest temperature, the best fits have the product of $n_{B}$ and $\sigma$ being about 2 s^−1^. In the absence of a more detailed model for the ^13^C–^13^C cross relaxation, we will fix $n_{B}$ and $\sigma$ at their average values of 1.6 and 0.9 s^−1^ respectively.

**Fig. 5:**
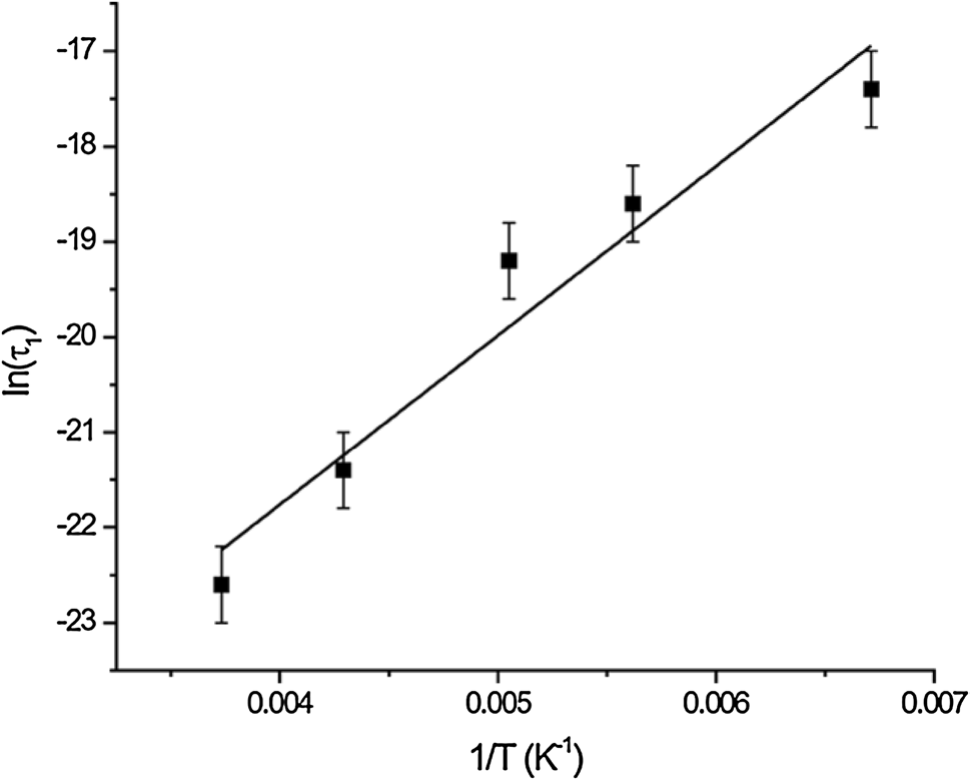
Arrhenius plot of the thermal activation of the methyl motion for the alanine in GAL. The least squares fit yields an activation energy of 3.5 kcal mol^−1^ ± 0.23 kcal mol^−1^ and an Arrhenius prefactor $\tau_{o}$ of 2.5 × 10^−13^ s ± 1.6 × 10^−13^ s

Having justified fixing $S^{2}, n_{B}$ and $\sigma$, the only remaining adjustable parameter in the model is the correlation time $\tau_{1}$. Using this as the only adjustable parameter, we then fit the recovery curves to find the best estimate of $\tau_{1}$ at each temperature. Figure 5 shows the Arrhenius plot for these simulations. From this plot we determine an activation energy of 3.5 kcal mol^−1^ ± 0.23 kcal mol^−1^ and an Arrhenius prefactor of 2.5 × 10^−13^ s ± 1.6 × 10^−13^ s. This activation energy is significantly greater than the values for the activation energy (2.8 kcal mol^−1^) of the alanines measured by Xue et al. (2007). It is also significantly lower than the value 5.35 kcal mol^−1^ for l-alanine measured by Batchelder et al. (1983). The difference between our observed value and the observed value of these two separate experiments can most likely be attributed to crystal packing. Our GAL tri-peptide would seem more akin to l-alanine than the SH3 domain of α-spectrin, because the alignment of the molecules in the crystal allows for intermolecular hydrophobic contacts between the leucines and the alanines. While the methyl groups may be packed tighter in GAL than in the hydrophobic core of an average protein, the bulky leucine sidechain prevents the molecule from packing as tightly as in the l-alanine crystal.

**Fig. 6:**
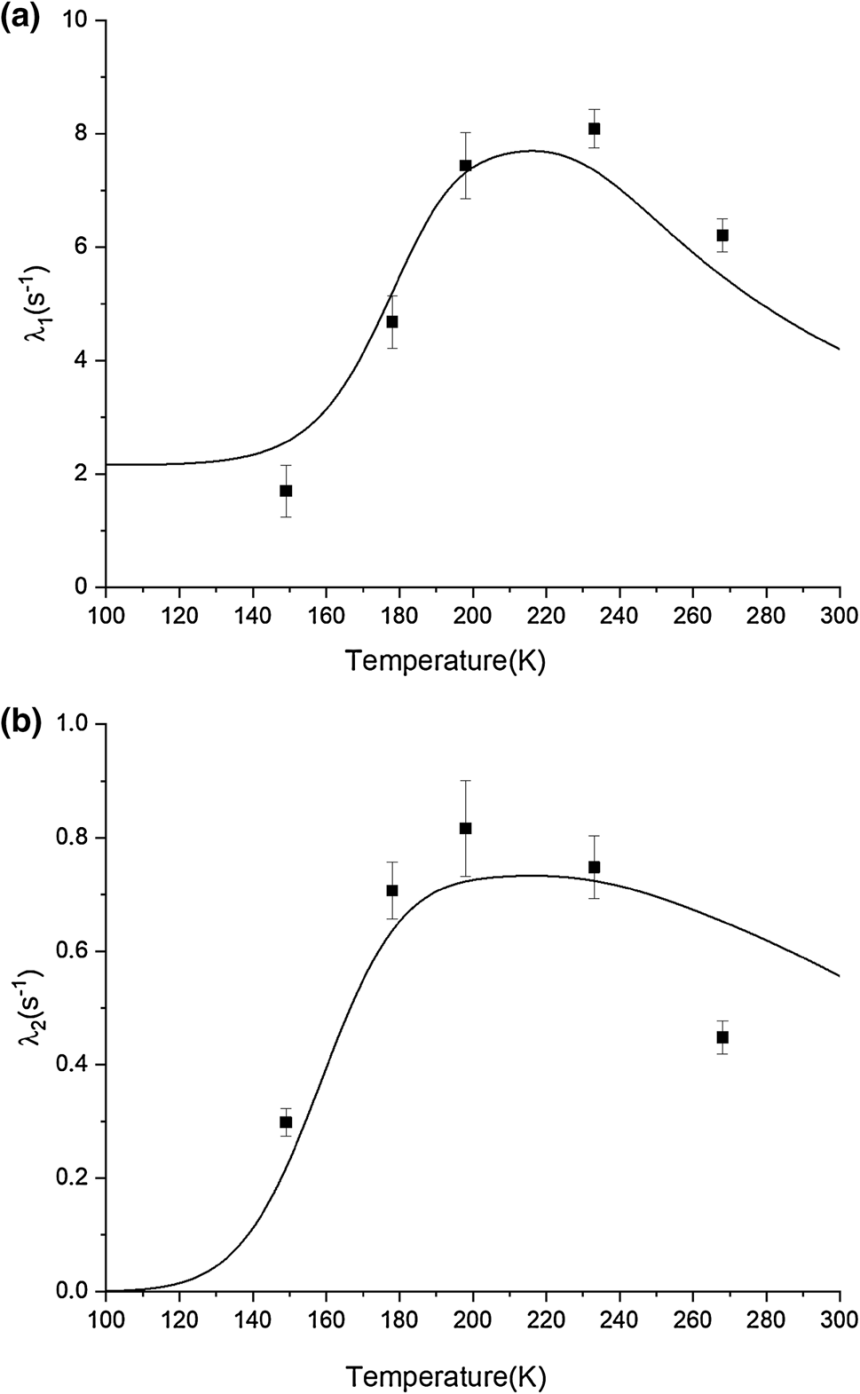
Comparison of the temperature dependence of the $R_{1}$ relaxation rates **a** fast and **b** slow for the alanine methyl taking into account spin diffusion to other ^13^C centers. The squares are the experimental data and the solid lines are calculated relaxation rates with the parameters $n_{B}, S^{2}, E_{a}, \tau_{o}\,{\text{and}}\,\sigma$ set to 1.6, 0.93, 3.5 kcal mol^−1^, 2.5 × 10^−13^ s and 0.9 respectively

Using the Arrhenius parameters extracted from Fig. 5, we can back calculate the temperature dependence of the slow and fast relaxation rates, and compare these curves with our experimental values. As shown in Fig. 6, the theoretical model agrees reasonably well with the theoretical data, especially in comparison to a model neglecting cross relaxation to other ^13^C sites. This model has improved on the predicted pre-exponential amplitudes, as shown in Table 5. However, the model still has difficulty predicting the pre-exponential amplitudes for the data points at 198 K and 178 K.

**Table 5:** Experimental and theoretical values for the parameter $$ \alpha $$ in the fitting function $$ M_{z} (t) = M_{0} (1 - \alpha e^{{ - \lambda_{1} t}} - (1 - \alpha )e^{{ - \lambda_{2} t}} ) $$ when setting $$ n_{B}, S^{2}$$, E_a_, $$ \tau_{o} $$ and $$ \sigma $$ set to 1.6, 0.93, 3.5 kcal mol^−1^, 2.5 × 10^−13^ s and 0.9 respectively

Temperature (K)	Experimental	Theoretical
268	0.58 ± 0.014	0.58
233	0.66 ± 0.014	0.71
198	0.60 ± 0.029	0.77
178	0.42 ± 0.034	0.70
149	0.22 ± 0.058	0.23

The remaining discrepancies can be from a variety of sources. One might infer from Fig. 5 that the barrier to rotation of the methyl group is temperature dependent, exhibiting a larger effective activation energy at the lower temperatures than it does at the higher temperatures. This can easily be justified as a result of the crystal contracting as the temperature is lowered. We have also assumed a temperature independent $S^{2}$, and perhaps most importantly a temperature independent ^13^C–^13^C cross relaxation rate. The fits reported in Tables 3 and 4 would indicate that the cross relaxation has a significant temperature dependence. Another possible source of errors in determining the activation parameters is uncertainty in the sample temperature. Our temperature is calibrated using solid–liquid phase transitions of a static sample. In addition, it was determined through the measurement of the water chemical shift on a sample of ubiquitin that there is a 10° difference between the sample temperature and the measured temperature when spinning at 22 kHz. Therefore, a constant of 10° has been added to the monitored temperature to account for heating from MAS. However, it is not certain that this differential will be constant across all temperatures as the density of the air around the sample is greater at lower temperatures.

A further likely explanation though is that the model used here is just too simple, and this injects systematic errors in the derived parameters. There is clearly ^1^H–^13^C cross relaxation occurring, and it will also be temperature dependent. Separating this component from the spin diffusion contribution experimentally would require relaxation data that could be convincingly fit better by a triple exponential recovery as opposed to a double exponential recovery, and this is unlikely to be obtained. Therefore there is a high degree of correlation between these derived parameters.

In spite of the shortcomings of the model and measurements just discussed, the framework described herein provides a theoretical model for the relaxation of a methyl group under MAS which is more accurate than others currently available. Given the dominant effect of spin diffusion on relaxation at longer times, it is expected that a more accurate characterization of molecular motions will be possible through the use of natural abundance ^13^C to suppress such effects, albeit it at the expense of sensitivity.

## Conclusions

A model for the ^13^C T_1_ relaxation of a methyl group in a powdered solid under MAS has been described which provides a quantitative accounting for the non-exponential relaxation recovery curves observed. The work of T&S has been extended to produce suitable powder averaged transition rates for a ^13^C^1^H_n_ spin system in analytic form, and these have been used in a rate matrix formalism to compute magnetization recovery curves. By comparing results for several isotopic variants of the same sample, it has been shown that in a ^13^C enriched sample, ^13^C–^13^C spin diffusion predominates the final stages of the return to equilibrium. Inclusion of this ^13^C–^13^C cross relaxation to a bath of ^13^C spins then is necessary if the T_1_ relaxation is to be modeled quantitatively even for a relatively rapidly relaxing methyl group. In the absence of spin diffusion the basic kinetics for the ^13^C spin–lattice relaxation is theoretically expected to be double exponential, where the slow rate primarily reflects the ^1^H–^13^C cross relaxation. When ^13^C–^13^C spin diffusion is included and dominates the^1^H–^13^C cross relaxation, the recovery is again effectively double exponential, but the slow time constant is now dominated by the spin diffusion rate, and does not reflect the molecular motion. Once this is recognized, it is possible to use the framework presented to quantitatively reproduce the relaxation rates experimentally encountered. This makes feasible the determination of the correlation time for the internal rotation of the methyl group and the associated activation parameters. The largest source of systematic error in determining these parameters in a ^13^C enriched sample appears to be the temperature dependence in the ^13^C–^13^C spin diffusion and the approximate manner in which spin diffusion is accounted for. Work using natural abundance samples may then provide more accurate measures of these parameters. In general the motions observed agree very well with those observed in solution NMR experiments.

In spite of the stated shortcomings, the successful modeling demonstrated here indicates that this approach will be a useful method for obtaining dynamics parameters from uniformly ^13^C enriched macromolecular systems. While this type of relaxation measurement will not compete in absolute accuracy with analysis of the temperature dependence of deuterium NMR powder patterns, it will be more readily applied in instances where site specific labeling is impractical.

While it is well known that ^1^H–^1^H spin diffusion to methyl groups can dominate ^1^H relaxation in organic solids, it is not as widely appreciated that ^13^C–^13^C spin diffusion can play as important a role, especially at MAS rates of 20 kHz or higher. Even at a moderately fast MAS rate, the ^13^C–^13^C dipolar couplings have not been completely attenuated, and can produce cross relaxation that competes with the limited relaxation pathways in a dynamically restricted solid. In MAS NMR studies of solid proteins, which nearly always make use of wholesale ^13^C enrichment, recognition of ^13^C–^13^C spin diffusion and accounting for it is a critical first step in drawing conclusions about molecular mobility on the basis of relaxation times. It is also interesting to note how the relaxation of other ^13^C, notably the CO and Cα centers, appear to be solely relaxed by spin diffusion to the ^13^C^1^H_3_ relaxation sinks. As noted in other studies of ^1^H relaxation, this could potentially be exploited to obtain distance information under suitable circumstances.

While the framework for computing relaxation of an X^1^H_n_ spin system in a powder sample under MAS was focused here on methyl groups, it could also be applicable to other situations. For many other geometries, whenever the motion in question can be approximated by the diffusion of the X–^1^H vector about a fixed axis at an angle $\varTheta$, the MAS and $\beta$ averaged transition rates in Eq. 13 may be found to provide accurate closed forms suitable for extracting dynamics parameters from NMR relaxation data.

## Electronic supplementary material

Below is the link to the electronic supplementary material.

Supplementary material 1 (PDF 131 kb)
