# Supplementary material for: Accounting for the temperature dependence of 13C spin–lattice relaxation of methyl groups in the glycyl–alanyl-leucine model system under MAS with spin diffusion
Source: J Biomol NMR. 2019 Aug 12;73(8):411–21. doi: 10.1007/s10858-019-00261-5 (PMC6817761; doi:10.1007/s10858-019-00261-5)
Supplement: Supplementary file 1 — Supplementary material 1 (PDF 131 kb) [file 10858_2019_261_MOESM1_ESM.pdf]

Supplemental A: Averaging methyl relaxation rates under MAS

From Torchia and Szabo [14], the ^{13}C relaxation rate for a methyl rotor is:

$$R_1^{SS} = \frac{9\omega_D^2}{64}\{g(\tau_1,\omega_I-\omega_s)A_1B_1+g(\tau_2,\omega_I-\omega_s)A_2B_2+g(\tau_1,\omega_I)2A_1B_4+g(\tau_2,\omega_I)2A_2B_5 \\ +g(\tau_1,\omega_I+\omega_S)4A_1B_5+g(\tau_2,\omega_I+\omega_S)A_2B_6\} \quad (1)$$

and from Macura and Ernst [17] the relaxation rates can be expressed as functions of their transition probabilities:

$$\begin{aligned} R_{II} &= 2(n_I-1)(W_1^{II}+W_2^{II})+n_S(W_0^{IS}+2W_1^{IS}+W_2^{IS}) \\ R_{IS} &= n_I(W_2^{IS}-W_0^{IS}) \\ R_{SS} &= 2(n_S-1)(W_1^{SS}+W_2^{SS})+n_I(W_0^{IS}+2W_1^{IS}+W_2^{IS}) \\ R_{SI} &= n_S(W_2^{IS}-W_0^{IS}) \end{aligned} \quad (2)$$

From here we can see that the zero, single and double quantum transition probabilities can be extracted as:

$$\begin{aligned} W_0 &= g(\tau_1,\omega_I-\omega_s)A_1B_1+g(\tau_2,\omega_I-\omega_s)A_2B_2 \\ W_1 &= g(\tau_1,\omega_I)A_1B_4+g(\tau_2,\omega_I)A_2B_5 \\ W_2 &= g(\tau_1,\omega_I+\omega_S)4A_1B_5+g(\tau_2,\omega_I+\omega_S)A_2B_6 \end{aligned} \quad (3)$$

Where

$A_1 = \sin^2 2\Theta$	$B_1 = \sin^2 2\theta$	$B_4 = \cos^2\theta + \cos^2 2\theta$
$A_2 = \sin^4\Theta$	$B_2 = \sin^4\theta$	$B_5 = \sin^2\theta + 0.25\sin^2\theta$
$A_3 = \sin^3\Theta\cos\Theta$		$B_6 = 1+6\cos^2\theta+\cos^4\theta$

Table S1: Angular functions used in explicit formulation for T_1

In order to account for MAS, we recast the problem in terms of the MAS rate (υ_r) using the relation:

$$\cos\theta(t,\beta) = \sin\beta\sin\theta_m\cos\omega_r t + \cos\beta\cos\theta_m \qquad (4)$$

where θ_m is the magic angle. Using these trigonometric identities:

$$\sin 2\theta = 2\sin\theta\cos\theta$$

$$\sin^2\theta = 1-\cos^2\theta \qquad (5)$$

$$\cos 2\theta = 2\cos^2\theta - 1$$

Table S1 can be recast as:

$A_1 = \sin^2 2\Theta$	$B_1 = 4(\cos^2\theta - \cos^4\theta)$	$B_4 = 4\cos^4\theta - 3\cos^2\theta + 1$
$A_2 = \sin^4\Theta$	$B_2 = 1 - 2\cos^2\theta + \cos^4\theta$	$B_5 = 1 - \cos^4\theta$
$A_3 = \sin^3\Theta\cos\Theta$		$B_6 = 1 + 6\cos^2\theta + \cos^4\theta$

Since Θ defines the angle between an C-H bond vector and the C_{3v} axis, there is no time dependence with respect to MAS, we only have to deal with functions that contain θ. Using the fact that:

$$\sin\theta_m = \sqrt{\frac{2}{3}} \quad \text{and} \quad \cos\theta_m = \sqrt{\frac{1}{3}} \qquad (6)$$

we can rewrite the time dependence of $\cos\theta(t)$ as:

$$\cos\theta(t,\beta) = \sqrt{\frac{2}{3}}\sin\beta\cos\omega_r t + \sqrt{\frac{1}{3}}\cos\beta \qquad (7)$$

Similarly

$$\cos^2\theta(t,\beta) = \frac{2}{3}\sin^2\beta\cos^2\omega_r t + \frac{1}{3}\cos^2\beta + \frac{2\sqrt{2}}{3}\sin\beta\cos\beta\cos\omega_r t \qquad (8)$$

$$\cos^4\theta(t,\beta) = \frac{4}{9}\sin^4\beta\cos^4\omega_r t + \frac{4}{9}\sin^2\beta\cos^2\beta\cos^2\omega_r t + \frac{8\sqrt{2}}{9}\sin^3\beta\cos\beta\cos^3\omega_r t$$
$$+\frac{4\sqrt{2}}{9}\cos^3\beta\sin\beta\cos\omega_r t + \frac{1}{9}\cos^4\beta + \frac{8}{9}\sin^2\beta\cos^2\beta\cos^2\omega_r t$$

At this point we can average over a rotor period as the relaxation rate is much greater than rotor period. Knowing,

$$\frac{1}{2\pi}\int_0^{\upsilon_r}\cos\omega_r tdt = \frac{1}{2\pi}\int_0^{\upsilon_r}\cos^3\omega_r tdt = 0$$

$$\frac{1}{2\pi}\int_0^{\upsilon_r}\cos^2\omega_r tdt = \frac{1}{2} \qquad (9)$$

$$\frac{1}{2\pi}\int_0^{\upsilon_r}\cos^4\omega_r tdt = \frac{3}{8}$$

the rotor averaged values for time dependence of θ is:

$$\cos\theta(\beta) = \sqrt{\frac{1}{3}}\cos\beta$$

$$\cos^2\theta(\beta) = \frac{1}{3}\sin^2\beta + \frac{1}{3}\cos^2\beta = \frac{1}{3} \qquad (10)$$

$$\cos^4(\beta) = \frac{1}{6}\sin^4\beta + \frac{2}{3}\sin^2\beta\cos^2\beta + \frac{1}{9}\cos^4\beta$$

$A_1 = \sin^2 2\Theta$	$B_1 = 4\left(\cos^2\theta - \cos^4\theta\right)$ $= 4\left(\frac{1}{3} - \frac{1}{6}\sin^4\beta - \frac{2}{3}\sin^2\beta\cos^2\beta - \frac{1}{9}\cos^4\beta\right)$	$B_4 = 4\cos^4\theta - 3\cos^2\theta + 1$

$A_2 = \sin^4 \Theta$	$B_2 = 1 - 2\cos^2\theta + \cos^4\theta$ $= \frac{1}{3} + \frac{1}{6}\sin^4\beta + \frac{2}{3}\sin^2\beta\cos^2\beta + \frac{1}{9}\cos^4\beta$	$B_5 = 1 - \cos^4\theta$
$A_3 = \sin^3\Theta\cos\Theta$		$B_6 = 1 + 6\cos^2\theta + \cos^4\theta$

Averaging over β yields

$$\frac{1}{2}\int_0^\pi \cos\beta\sin\beta d\beta = 0, \quad \frac{1}{2}\int_0^\pi \cos^2\beta\sin\beta d\beta = \frac{1}{3}, \quad \frac{1}{2}\int_0^\pi \cos^2\beta\sin^3\beta d\beta = \frac{2}{15}$$

$$\frac{1}{2}\int_0^\pi \cos^4\beta\sin\beta d\beta = \frac{1}{5}, \quad \frac{1}{2}\int_0^\pi \sin^3\beta d\beta = \frac{2}{3}, \quad \frac{1}{2}\int_0^\pi \sin^5\beta d\beta = \frac{8}{15} \qquad (11)$$

Finally, we can write the angular functions for the explicit forms for T_1as :

$A_1 = \sin^2 2\Theta$	$B_1 = \frac{8}{15}$	$B_4 = \frac{4}{5}$
$A_2 = \sin^4\Theta$	$B_2 = \frac{8}{15}$	$B_5 = \frac{4}{5}$
$A_3 = \sin^3\Theta\cos\Theta$		$B_6 = \frac{16}{5}$

For the W_1^{II} and W_2^{II}, the transition probabilities are the same as the heteronuclear case, with the exception that Θ is now defined by the angles created by the C_{3v} axis and the H-H vector, which is 90 degrees.

Supplemental B: Relaxation profile corresponding to the correlation times extracted from 3-spin system type simulation.

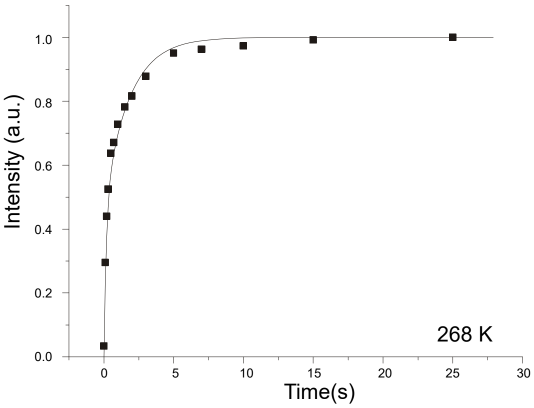

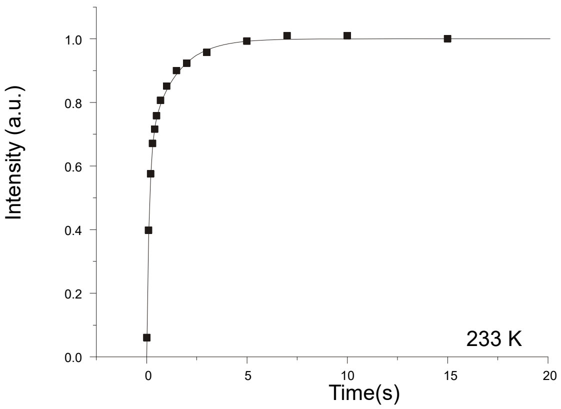

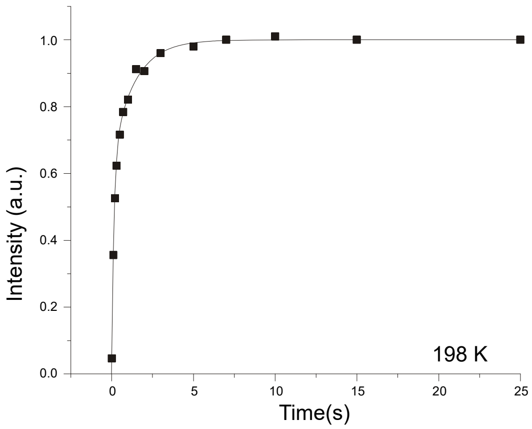

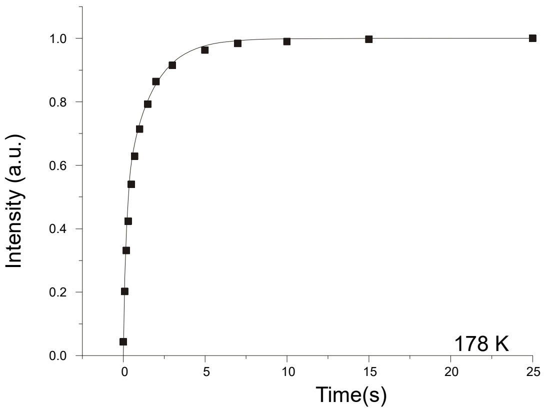

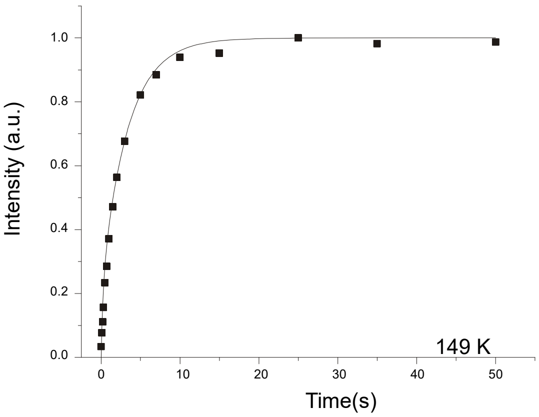

Supplemental Figure1 – Theoretically determined relaxation profile compared against experimentally determined relaxation profile. The squares are the experimental data and the solid lines are calculated relaxation rates with the parameters n, S^2, E_a, τ_o and σ set to 1.6, 0.93, 3.5 kcal/mol, 3.5×10^{-13} s and 0.9 respectively.
